# Clinical evaluation of the effectiveness of fusion‐induced asymmetric transcription assay‐based reverse transcription droplet digital PCR for *ALK* detection in formalin‐fixed paraffin‐embedded samples from lung cancer

**DOI:** 10.1111/1759-7714.13535

**Published:** 2020-06-16

**Authors:** Yuanyuan Liu, Shafei Wu, Xiaohua Shi, Linping Lu, Lingxiang Zhu, Yong Guo, Li Zhang, Xuan Zeng

**Affiliations:** ^1^ Department of Pathology Peking Union Medical College Hospital, Molecular Pathology Research Center, Chinese Academy of Medical Sciences Beijing China; ^2^ TargetingOne Corporation Beijing China; ^3^ National Research Institute for Family Planning Beijing China; ^4^ Department of Biomedical Engineering School of Medicine, Tsinghua University Beijing China; ^5^ Department of Respiratory Diseases Peking Union Medical College Hospital, Chinese Academy of Medical Sciences Beijing China

**Keywords:** *ALK*, asymmetric, droplet digital PCR, lung cancer, rearrangement

## Abstract

**Background:**

Accurate detection of anaplastic lymphoma kinase (*ALK*) rearrangement is the prerequisite for anti‐*ALK* therapy for the patient with non‐small cell lung cancer (NSCLC). Fusion‐induced asymmetric transcription assay (FIATA)‐based reverse transcription droplet digital PCR (RT‐ddPCR) was developed and performed for *ALK* status survey in NSCLC samples.

**Methods:**

A total of 269 cases of formalin‐fixed paraffin‐embedded (FFPE) specimens from NSCLC, in which *ALK* status was confirmed by both fluorescence in situ hybridization (FISH) and immunohistochemistry (IHC), were analyzed by FIATA‐based RT‐ddPCR.

**Results:**

In the *ALK*‐positive group, the 3′ *ALK* transcript copies range was 336.6–107 955.4, and the R3 [(the ratio of the 3′ *ALK* transcript copy numbers to the internal reference gene transcript copy numbers) × 100] was 17.23–672.77. In the *ALK*‐negative group, the 3′ *ALK* transcript copies range was 3.7–1370.6, and the R3 range was 0.10–15.57. The lowest R3 level in the *ALK*‐positive group was significantly higher than the highest R3 level in the *ALK*‐negative group. A positive correlation between the proportion of cancer cells in the tissue section and *ALK* RNA expression level (R3) was found (*P* < 0.05). There was no relationship between the percentage of FISH positive cells or FISH positive signal patterns and R3 level of the *ALK* gene. Compared with FISH and IHC, the clinical sensitivity and specificity of FIATA‐based RT‐ddPCR for *ALK* detection were 100%, respectively.

**Conclusions:**

An absolute quantitative FIATA‐based RT‐ddPCR was developed and validated for *ALK* fusion detection in NSCLC. This method can rapidly, accurately, and objectively classify *ALK* types and help with individual therapy.

## Introduction

Carcinogenic anaplastic lymphoma kinase (*ALK*) gene rearrangement occurs in approximately 5%–7% of non‐small cell lung cancer (NSCLC) patients. With the clinical application of inhibitors targeting activated *ALK*, such as crizotinib, ceritinib, and alectinib, the survival rate of patients with NSCLC has increased remarkably.^1^, ^2^, ^3^ To date, at least 19 *ALK* rearrangement partner genes have been identified, including echinoderm microtubule‐associated protein‐like 4 (*EML4*), kinesin family member 5B (*KIF5B*), kinesin light chain 1 (*KLC1*), TRK‐fused gene (*TFG*), and translocated promoter region (*TPR*) etc. resulting from chromosomal inversion or translocation. More than 30 *EML4‐ALK* fusion variants have been found.^4^

Classical methods for gene variation detection, such as fusion specific‐based polymerase chain reaction (PCR) (eg, traditional reverse transcription quantitative PCR, RT‐qPCR) and/or DNA sequencing (eg, next generation sequencing, NGS), are greatly limited for *ALK* assessment in daily practice mainly because of extremely diverse and complex *ALK* fusion patterns. For example, some loci of fusion gene uncovered or unknown may be missed.^5^, ^6^, ^7^ In addition, preferred methods for *ALK* detection must be suited to formalin‐fixed paraffin‐embedded (FFPE) and biopsy samples because most NSCLC patients are diagnosed in the advanced stage, and tumor tissue is difficult to obtain or limited. This was a key point for acquiring an accurate result with a minimum of specimens in the real world. Therefore, fluorescence in situ hybridization (FISH, using a Vysis *ALK* Break Apart FISH Probe Kit) and immunohistochemistry (IHC, using Ventana *ALK* D5F3 platform) are mainly recommended by the Molecular Testing Guidelines for the Selection of Lung Cancer Patients for Treatment with Targeted Tyrosine Kinase Inhibitors from the College of American Pathologists (CAP), the International Association for the Study of Lung Cancer (IASLC), the Association for Molecular Pathology (AMP), and the expert consensus on clinical practice of *ALK* fusion detection in NSCLC in China, respectively, because of their prominent superiorities involving rapid turnaround time (1–2 day), consumption of 1–2 slides only, and regardless of partner gene and *ALK* variant patterns.^8^, ^9^

However, the inherent disadvantages of FISH and IHC assays for *ALK* detection have also been previously described. FISH has not been considered a primary option for *ALK* detection, especially in extensive basic hospitals in China, because of the high cost, necessary professional training for interpretation, and challenge of identification with the subtle fluorescent signals under dark fields, as well as hidden break‐apart signals because of multiple rearrangements in the genome or *ALK* fusion without downstream pathological products as a consequence of complicated genetic events.^10^, ^11^, ^12^, ^13^ IHC has been the primary method for *ALK* screening because of its great popularity and low cost in China. However, deviation of the results and missed cases have been observed because of subjective interpretation and lack of strong immunoreactivity (affected by the composition of mucus) in some specimens. Furthermore, IHC results may not be accurate due to inappropriate handling of tissues during preanalytical and analytical phases, such as formalin fixation, antigen retrieval, and immunostaining.^14^, ^15^

There are 29 exons in the *ALK* gene. Activation of *ALK* in NSCLC is usually caused by a DNA strand break between exons 19 and 20, followed by a fusion of the segment containing kinase domain after the exon 20 with the partner gene.^16^, ^17^ In cases where the 5′ *ALK* fragment has been lost but the 3′ *ALK* fragment was maintained when the break happened, carcinogenic *ALK* fusion with partner genes could still be triggered due to the intact sequences encoding *ALK* kinase domain and lead to *ALK* activation (isolated 3′ red signals of FISH positive pattern can be confirmed by protein or RNA expression testing).^18^ Theoretically, *ALK* rearrangements, including all of the known and unknown fusion patterns, can be detected by measuring the increased transcript levels after exon 20, which is a similar method to IHC and FISH assay. A sensitive approach for *ALK* detection using NanoString nCounter technology has been developed and validated which targets both *ALK* 3′ overexpression and common fusions despite the high cost of the equipment.^19^, ^20^

In the current study, a fusion‐induced asymmetric transcription assay (FIATA)‐based reverse transcription droplet digital PCR (a new RT‐ddPCR system, TD‐1) was performed for *ALK* detection. A total of 269 FFPE samples from 89 *ALK*‐positive and 180 *ALK*‐negative patients with NSCLC were analyzed for inspecting the sensitivity and specificity of the method. Subsequently, the detectability of the method for 57 cases of different FISH positive types was assessed in detail since FISH is recognized as the gold standard for *ALK* detection and a comparator for the other approaches. Our study will be helpful for recognizing the patients with NSCLC who may potentially benefit from a variety of *ALK* inhibitors.

## Methods

### Patient cohort

A total of 269 FFPE samples, including 89 cases of *ALK* positive (70 surgical and 19 biopsy) and 180 cases of *ALK* negative (150 surgical and 30 biopsy) (double confirmed by IHC and FISH), from NSCLC patients acquired between August 2018 and October 2019 archived in Peking Union Medical College Hospital were collected. This retrospective study was approved by the institutional review board of Peking Union Medical College Hospital.

### IHC for *ALK* protein expression detection

IHC assay was performed according to the manufacturer's instructions. In brief, 4 μm thick tissue slides were stained with the D5F3 rabbit monoclonal primary antibody on a Ventana BenchMark Ultra autostainer with the OptiView DAB IHC Detection Kit and OptiView Amplification Kit (Ventana Medical Systems, Inc., Tucson, AZ, USA) (D5F3). Binary scoring (positive or negative) to interpret IHC staining results was done. A positive result of *ALK* status was determined for the case with strong granular cytoplasmic staining in any percentage of tumor cells. In contrast, the absence of strong granular cytoplasmic staining in the tumor cells was classified as a negative result based on the guidelines.^21^

### FISH for *ALK* gene fusion detection

A 4 μm FFPE tissue section was applied to FISH assay with the Vysis *ALK* Break Apart FISH Probe Kit (Abbott Molecular, Chicago, IL, USA) using the ThermoBrite Elite Automated FISH slide preparation system (Leica, Biosystem, Buffalo Grove, IL, USA) according to the manufacturer's protocol. The FISH slide was scanned using the CytoVision DM6000B fluorescent microscope system (Leica, Biosystem, Buffalo Grove, IL, USA). At least 50 nonoverlapping nuclei of tumor cells were counted, as well as the 3′ signals (labeled by SpectrumOrange), 5′ signals (labeled by SpectrumGreen), and fusion signals were scored, respectively. A case was considered as *ALK*‐positive if at least 15% of the tumor cells had either split red and green signals ≥2 signal diameter and/or an isolated red signal (green signal deletion) following the *ALK* FISH interpretation criteria.^22^

### FIATA‐based RT‐ddPCR for *ALK* mRNA detection

RNA was extracted from 2–3 surgical or 6–8 biopsy FFPE tissue slides of 4–5 μm thickness using a RNeasy FFPE Kit (Qiagen, Hilden, Germany) and quantified and qualified using a NanoDrop One spectrophotometer.

*ALK* status was analyzed using a FIATA *ALK* testing kit (TargetingOne, Beijing, China). Two primers were designed with sequences located on exons 22–23 for the 3′ portion, exons 17–18 for the 5′ portion of *ALK* gene, and internal control (IC) gene (Abelson murine leukemia viral oncogene homolog 1, *ABL1*), respectively. The primer sequence located on exons 2–3 was used for the case with atypical *ALK* results after being tested with the conventional primers above (ie, 3′ transcript increment resulting from *ALK* fusion rather than a full length *ALK* amplification was identified) (Table 1). A 30 μL reaction mixture (the components are listed in Table 1) was prepared for the emulsion generation using TargetingOne Drop Maker M1 (TargetingOne, Beijing China), and approximately 60 000 droplets (0.3 nL per droplet volume containing a signal RNA molecule) were obtained.

**Table 1:** PCR reaction system

Gene	Exon	Primer (5′ → 3′)	Located on cDNA	Amplicon length
*ALK*	E22‐23	Forward: TCTGAACAGGACGAACTGGA	3469–3488	62 bp
		Reverse: TGGTGGTTGAATTTGCTGA	3512–3530	
		Probe: FAM‐CTCATGGAAGCCC‐MGB	3493–3505	
	E17‐18	Forward: CAGTCCACTGGGCATCCT	2877–2894	56 bp
		Reverse: CCCCGTGGCCTTCCAT	2917–2932	
		Probe: FAM‐CCCCAGCTTTAAAAG‐MGB	2900–2914	
	E2‐3	Forward: ATCTCACCTGGATAATGAAAGACT	1638–1661	75 bp
		Reverse: CAAAGCTGCACTCCAGACC	1694–1712	
		Probe: FAM‐CTTTCCTGTCTCATCG‐MGB	1668–1683	
*ABL1*	E2‐3	Forward: TGGAGATAACACTCTAAGCATAACTAAAGGT	418–449	81 bp
		Reverse: GCTTCACACCATTCCCCATTGT	477–498	
		Probe: VIC‐AAGCTCCGGGTCTTAG‐MGB	452–467	

**Components**	
Total volume	30 μL
SuperMix	9 μL
Enzyme mix	3 μL
3′/5′ *ALK* PCR solution (containing primers)	3 μL
Template RNA	10–300 ng
RNase free ddH_2_O	To reach total volume of 30 μL

After 55°C for 15 minutes and 95°C for 10 minutes, the total 40 cycles of PCR reaction were carried out in Bio‐Rad PTC200 thermal cycler according to the following procedure: 94°C for 30 seconds, 57°C for one minute, and 12°C cooling for five minutes. The PCR product was then loaded onto Chip Reader R1 (TargetingOne, Beijing, China) for detecting the FAM (488 nm laser) and VIC (532 nm laser) fluorescence intensity for each droplet.

The cluster plots were calculated using TargetingOne analysis software (TargetingOne, Beijing China). The 3′ and 5′ of *ALK* RNA transcripts copies (FAM) and *ABL1* RNA transcript copies (VIC) were counted, respectively. R3 ([the ratio of the 3′*ALK* transcript copy numbers to the internal reference gene transcript copy numbers] × 100) and R5 ([the ratio of the 5′*ALK* transcript copy numbers to the internal reference gene transcript copy numbers] × 100) were measured, respectively. R3 represented the expression level of the *ALK* gene. The cutoff value of R3 was defined as 16.40 as we described in the previous study.^23^

### Statistical analysis

The data analysis was implemented using SPSS version 22.0 and GraphPad Prism 8.0. Correlations were evaluated using Spearman's rank correlation coefficients. Significance was accepted as *P*‐value <0.05.

## Results

### Sensitivity and specificity of *ALK* status detection by FIATA‐based RT‐ddPCR

**Figure 1:**
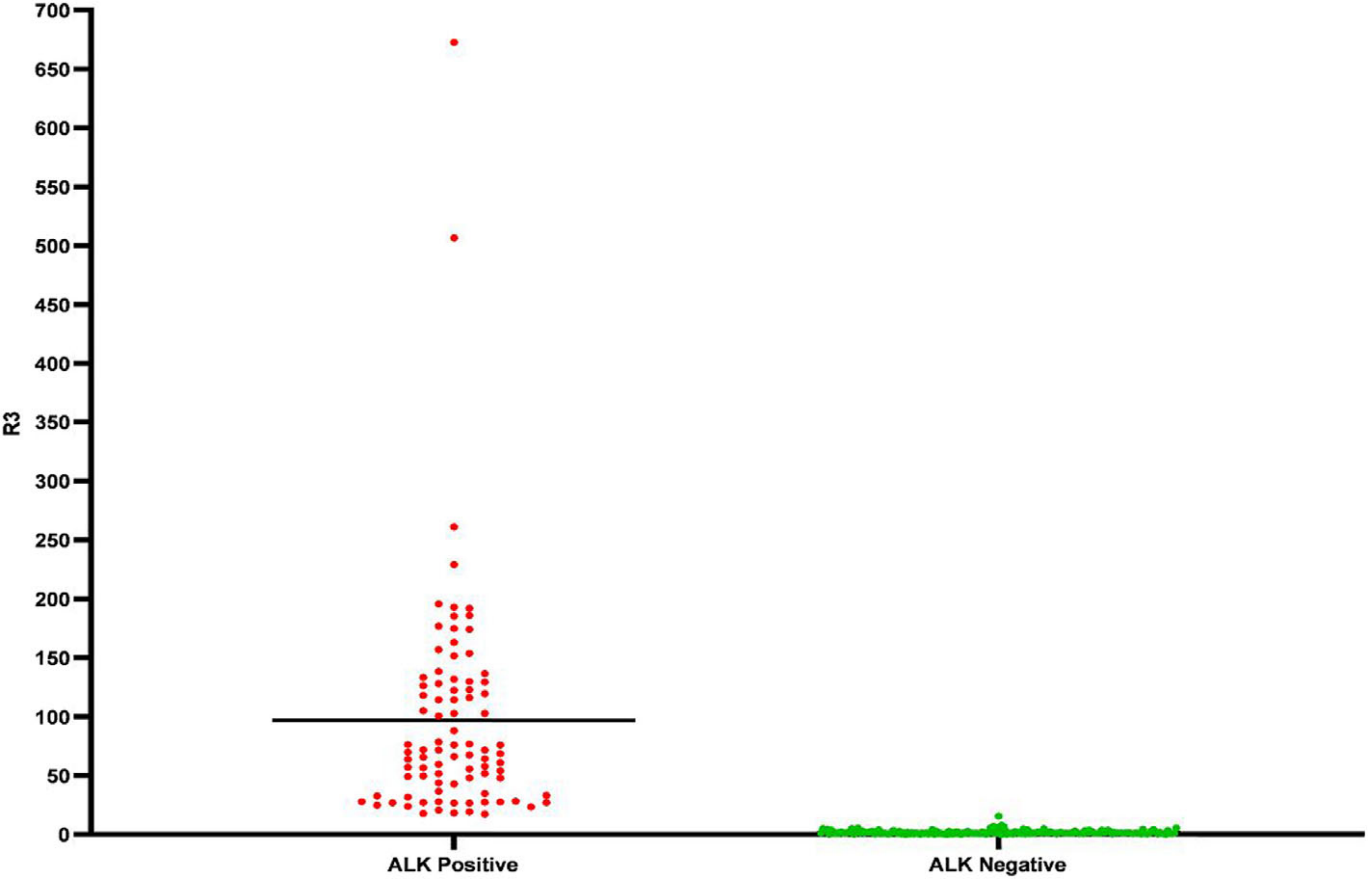
*ALK* mRNA expression levels (R3) in our series including 89 *ALK*‐positive and 180 *ALK*‐negative samples from NSCLC. (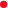) *ALK*‐positive; (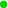) *ALK*‐negative.

**Figure 2:**
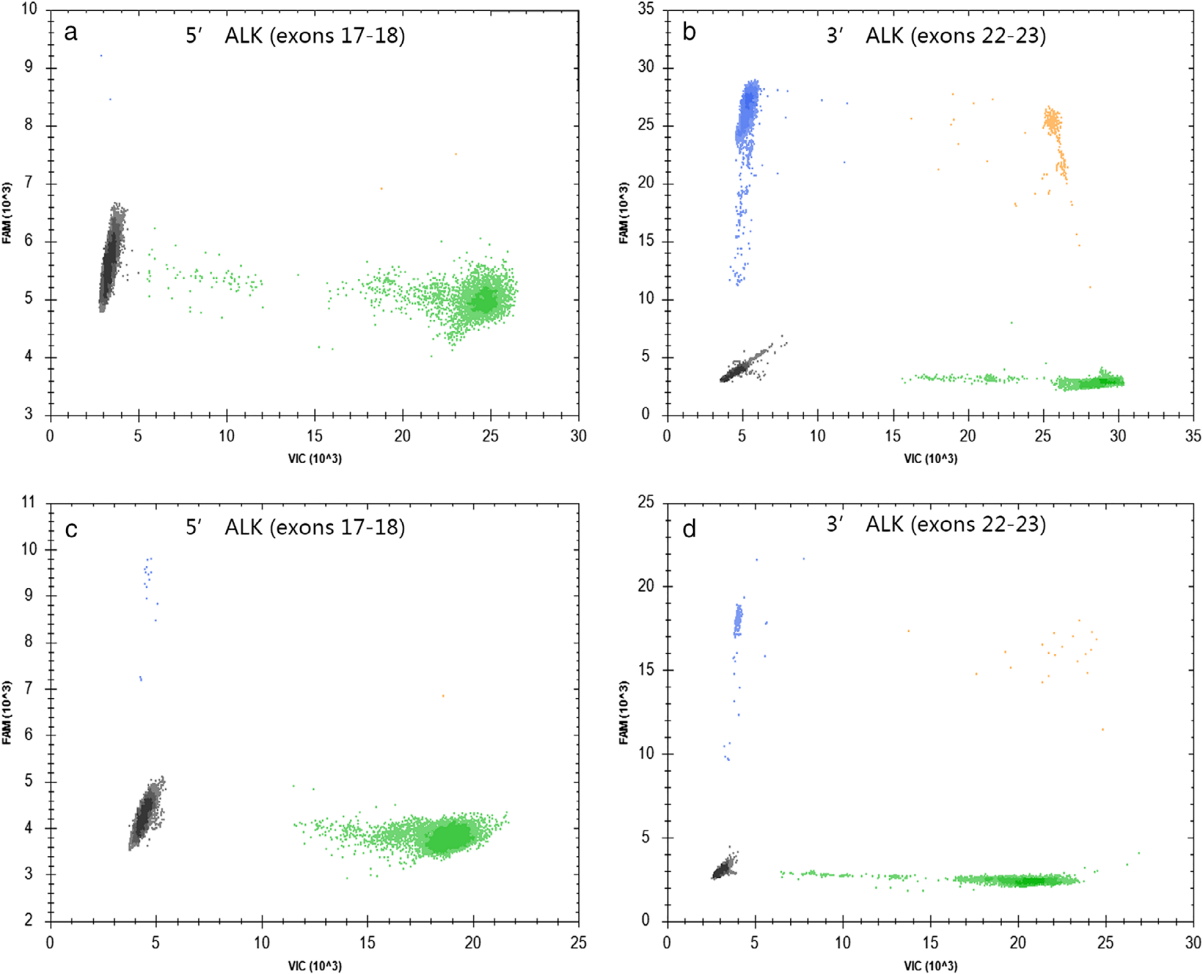
*ALK* RNA expression detected by FIATA‐based RT‐ddPCR with representative graphs from *ALK*‐positive Case 10: (**a**) R5 0.21; (**b**) R3 102.84; and from *ALK*‐negative Case 81 (**c**) R5 0.30; (**d**) R3 4.65. (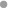) FAM^−^/VIC^−^; (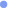) FAM^+^/VIC^−^; (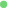) FAM^−^/VIC^+^; (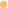) FAM^+^/VIC^+^.

**Figure 3:**
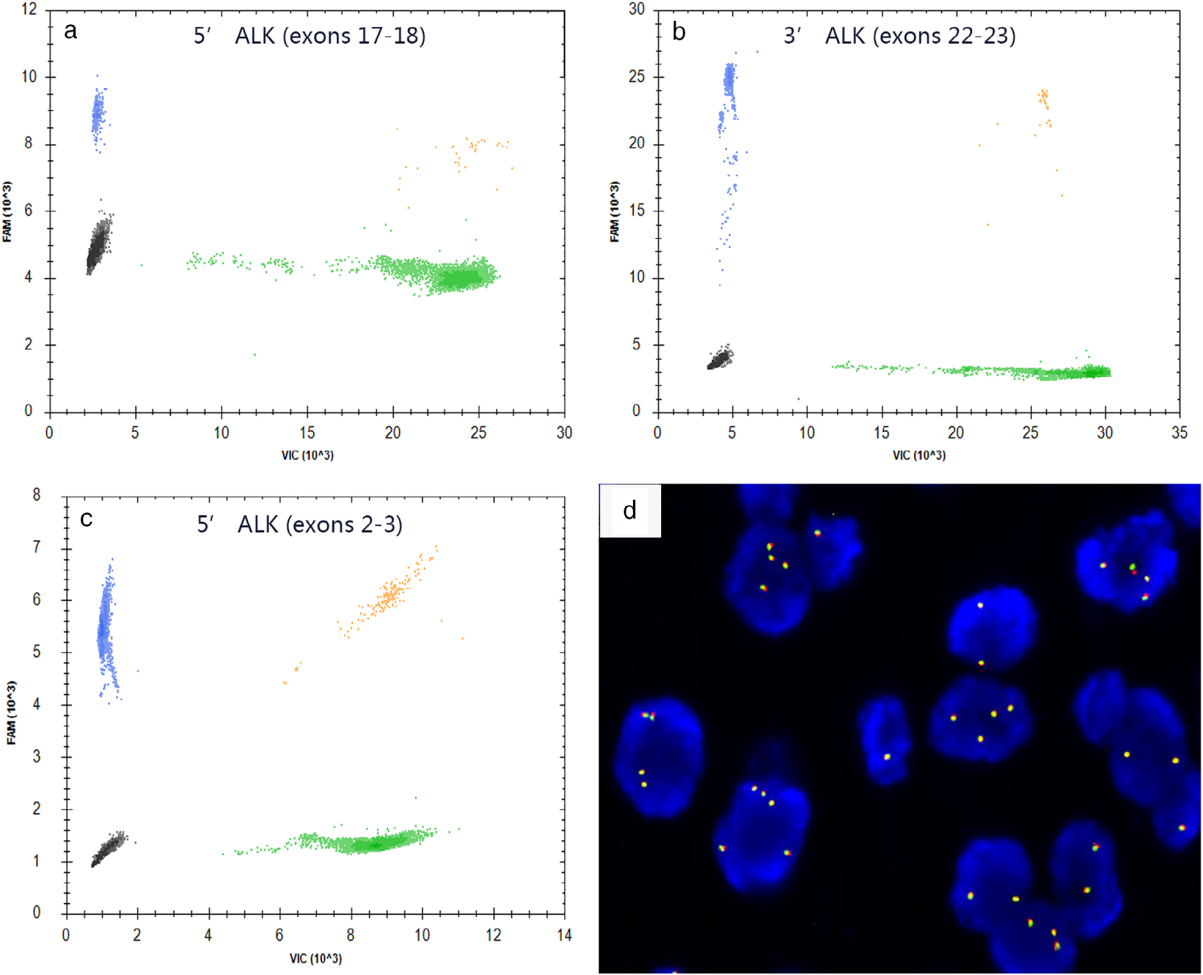
*ALK* RNA expression detected by FIATA‐based RT‐ddPCR in Case 94. (**a**) Exons 17–18 of 5′*ALK* expression (R5) were 13.44. (**b**) Exons 22–23 of 3′*ALK* expression (R3) were 15.57. (**c**) Exons 2–3 of 5′*ALK* expression were 66.21. (**d**) FISH‐negative with fusion signal pattern in tumor cells. (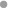) FAM^−^/VIC^−^; (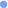) FAM^+^/VIC^−^; (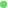) FAM^−^/VIC^+^; (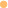) FAM^+^/VIC^+^.

**Figure 4:**
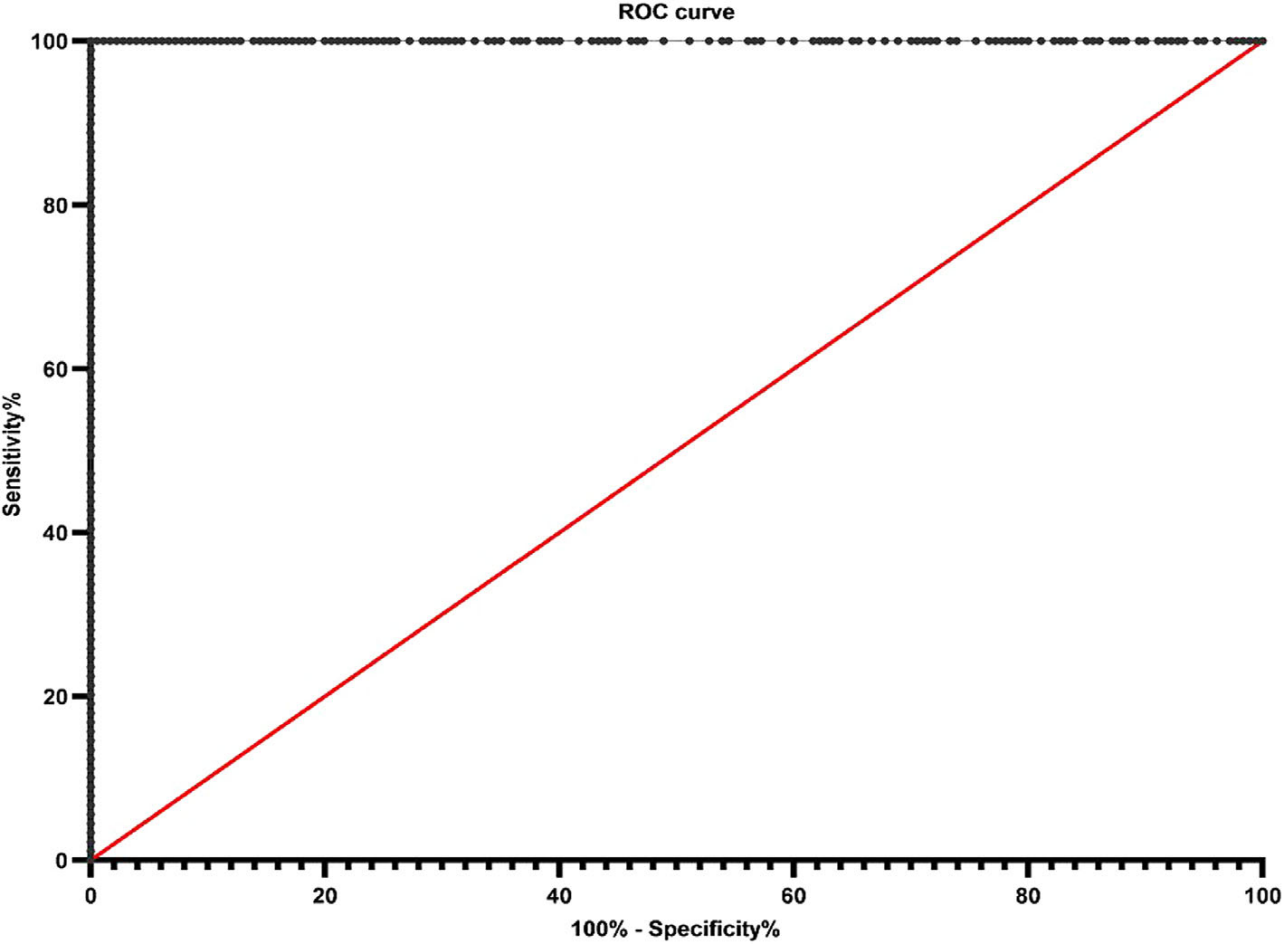
The receiver operator characteristic (ROC) curve for sensitivity and specificity of *ALK* detection using FIATA‐based RT‐ddPCR in the present study.

*ALK* RNA expression was measured by TD‐1 RT‐ddPCR with FIATA strategy for 269 cases of NSCLC including 89 *ALK*‐positive and 180 *ALK*‐negative specimens. In the *ALK*‐positive group, the 3′ *ALK* transcript copies range was 336.6–107 955.4, the 5′ *ALK* transcript copies range was 0.0–7924.8, the R3 range was 17.23–672.77, and the R5 range was 0.00–44.53. In the *ALK*‐negative group, the 3′ *ALK* transcript copies range was 3.7–1370.6, the 5′ *ALK* transcript copies range was 0.0–697.9, the R3 range was 0.10–15.57, and the R5 range was 0.00–13.44 (Fig 1, 2). The lowest R3 level in the *ALK*‐positive group was significantly higher than the highest R3 level in the *ALK*‐negative group. *ALK* gene was ulteriorly analyzed using primer covering exons 2 to 3 for case 94 with obviously higher R3 and R5 simultaneously (R3 of exons 22–23 was 15.57 and R5 of exons 17–18 was 13.44, respectively) than others in the same *ALK*‐negative group (R3 0.10–7.92; R5 0.00–2.72). The R5 of exons 2–3 of the *ALK* gene was 66.21 in case 94 (Fig 3). Compared with *ALK* status assessed by FISH and IHC, the clinical sensitivity and specificity of FIATA‐based RT‐ddPCR were 100%, respectively (89/89, 95% confidence interval, 94.8%–100%; 180/180, 95% confidence interval, 97.4%–100%.) (Fig 4). The accuracy of the assay was 100% (269/269). Template RNA content was between 14.2 ng and 397.5 ng. The association between the *ALK* RNA expression (R3 level) and the content of template RNA was not found in our cohort.

### Evaluation of *ALK*‐positive cases with different FISH patterns by FIATA‐based RT‐ddPCR

**Figure 5:**
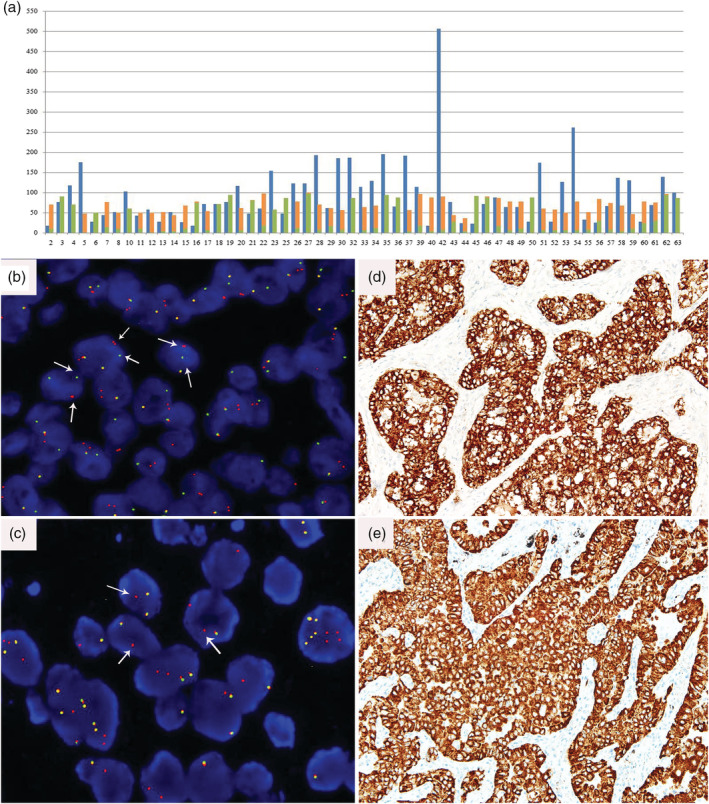
(**a**) *ALK* RNA expression (R3 level) detected by FIATA‐based RT‐ddPCR with corresponding percentage of FISH‐positive cells and proportion of different FISH‐positive signal patterns in 57 *ALK* positive cases. x‐axis, sample numbers; y‐axis, percentage of tumor cells with BA or IR signal pattern, or R3 level (**b**) *ALK* FISH signals with BA pattern predominates in Case 39 and (**c**) IR pattern in Case 32, as well as their (**d** and **e**) protein overexpression detected by IHC are shown, respectively. BA, break apart; IR, isolated red. (**a**) (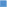) R3; (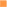) BA; (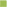) IR.

A total of 57 cases of *ALK* FISH‐positive with different signal patterns, which included 37 cases with dominant break apart (BA) signals and 20 cases with dominant isolated red (IR) signals, selected from our cohort were analyzed by FIATA‐based RT‐ddPCR. The tumor cells were between 5% and 90% in FFPE sections of our *ALK*‐positive cohort. Case 42 had the highest R3 level (506.63), and 20% of tumor cells in the FFPE slide harbored 90% FISH‐positive cells in the slide (a dominant BA signal pattern). Case 2 had the lowest R3 level (17.23), and 10% of tumor cells in the FFPE slide harbored 70% FISH‐positive cells in the slide (also a dominant BA signal pattern). Case 4, with the maximum of tumor cells in the sample (90%), had an R3 of 118.16 with 70% FISH‐positive cells (100% IR). Case 57, with a minimum of tumor cells in the slide (5%), had an R3 of 66.25 with 74% FISH‐positive cells (including 89% BA and 11% IR). The proportion of FISH‐positive cells was between 36% and 100% in this population. Case 27, with 100% FISH‐positive cells (2% BA, 98% IR) in the slide, possessed an R3 of 123.11 with 25% tumor cells. Case 44, with the lowest percentage of FISH‐positive cells (36%, including 32% BA and 4% IR) in the slide, had an R3 of 23.98 with 10% tumor cells (Fig 5). The correlation between FISH‐positive signal patterns or the proportion of FISH‐positive cells and *ALK* RNA expression was not found.

**Figure 6:**
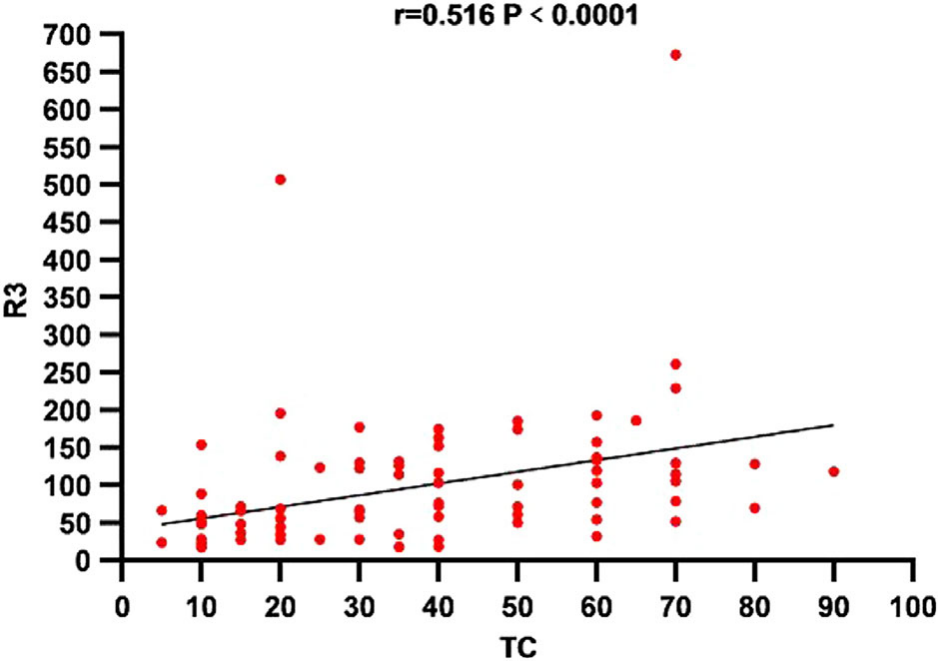
The correlation of *ALK* RNA expression (R3 level) detected by FIATA‐based RT‐ddPCR and the corresponding percentage of tumor cells (TC) were exhibited in 89 cases of *ALK*‐positive samples.

A total of 89 cases were *ALK*‐positive, and there was a relationship between the proportion of cancer cells in the samples and the *ALK* R3 level (*P* < 0.05) (Fig 6).

## Discussion

In addition to IHC and FISH, a number of commercial kits for *ALK* RNA transcript‐based RT‐qPCR assay, which show high degrees of concordance with FISH and IHC, have been developed and used in clinical laboratories.^24^, ^25^ Although the common examination methods based on PCR discloses the exact variants of *ALK* fusion, a large number of samples are required because multiple PCR reactions are required to detect diverse *ALK* aberrations. However, *ALK* fusion variations with undiscovered rare partners are unlikely to be discerned by this approach depending on fusion loci designing.^26^

Reasonably, all various forms of pathogenic *ALK* rearrangement could be captured by FIATA‐based RT‐ddPCR irrespective of fusion points and upstream fusion partners with *ALK* gene. Lung *et al*. determined the *ALK* status of NSCLC samples and further identified *ALK* false negative from FISH or IHC detection using an established TaqMan‐based RT‐qPCR assay with imbalanced excogitation of RNA transcript levels between upstream and downstream of the *ALK* gene. The assay was more effective at discriminating *ALK* rearrangement from overexpression according to the data. The identical percentage of *ALK*‐positive patients with NSCLC was detected by FISH and RT‐qPCR with 5′/3′ imbalance strategy, which was developed by the researchers from another study. Meanwhile, *ALK* fusion in circulating tumor RNA (ctRNA) was recognized via the method.^27^, ^28^

In the present study, we developed and validated a novel quantitative and efficient FIATA‐based RT‐ddPCR technique (TD‐1 RT‐ddPCR system, expatiated in previous studies^29^, ^30^). Based on 3′/IC asymmetric assay, *ALK* status of 269 samples from NSCLC was analyzed by the approach. Compared with FISH and IHC, 100% clinical sensitivity, specificity, and accuracy were revealed, respectively. Its comparable capability for *ALK* estimation in NSCLC as FISH and IHC was justified. Additionally, the method can be easily adopted and integrated into daily work flow due to its easy operation, short turnaround time, and automatically generating objective result. Particularly, the *ALK* status was accurately distinguished from conventional 4 μm thickness of 2–3 surgical or 6–8 biopsy tissue sections with as low as 5% tumor cells/slide dependent on high sensitivity and specificity of the assay. Beyond that, the total template RNA content of our samples from the real world was 14.2 ng–397.5 ng, all of which were suitable for *ALK* detection and the accurate and consistent results with FISH and IHC were acquired. This is favorable due to the limited availability of biopsy tissue which is required for clinical diagnosis, including molecular typing for NSCLC, most of which was advanced disease and difficult to sample. Moreover, FFPE samples were prepared and archived within one year by routine clinical procedures in this study. The *ALK* status of all samples was confirmed via FIATA‐based RT‐ddPCR and the RNA integrity‐related issue was disregarded. Our data suggested that this new method for *ALK* detection possessed high sensitivity and tolerance for the clinical samples.

In our cohort, the RNA expression of the 5′ *ALK* portion was higher than other cases in the *ALK*‐negative group in addition to its 3′ *ALK* portion amplification for case 94 (R3 15.57 and R5 13.44, respectively). Subsequently, we inspected the upstream of RNA transcript *via* amplicon from exons 2–3 of the *ALK* gene. The R5 of exons 2–3 was 66.21; thus, the full length of *ALK* gene amplification rather than oncogenic *ALK* rearrangement was realized (concordant with FISH and IHC results). This case was also further confirmed and categorized *ALK* as negative via NGS assay (data not shown). The mechanism and biological sense of this rare endogenous 5′ RNA transcript amplification was not clear in NSCLC. *ALK* status was tested in 30 cases of peritumoral normal tissues in our previous study. On the other hand, the R3 and R5 in benign samples were lower than those in the *ALK*‐negative group.^23^ The good specificity of FIATA‐based RT‐ddPCR was also verified. Our results implied that R3 can present the *ALK* status and is used as a potential alternative tool for *ALK* evaluation.

In our cohort, based on the total RNA extracted from the whole tissue section (it was easier to operate for nonmicrodissection in the clinical laboratory) and proportion of tumor cells on the slides estimated by area, there was no correlation between the percentage of *ALK*‐positive cells detected by FISH and *ALK* RNA expression level detected by FIATA‐based RT‐ddPCR, which differed from the findings in previous relevant studies (ie, the positive correlation between the number of *ALK* fusion cells by FISH and RNA or protein expression by qPCR or IHC^28^, ^31^). FISH‐positive/IHC‐negative and FISH‐negative/IHC‐positive cases responding to *ALK* inhibitors have been reported in NSCLC,^32^, ^33^ and two NSCLC patients with <15% fusion cells by FISH but low copy numbers of *EML4‐ALK* fusion by qPCR responded well to crizotinib after failure of chemotherapy was described.^34^ Therefore, PCR‐based *ALK* detection assay was more sensitive than conventional methods. Compared with semi‐quantitative and manual‐scoring FISH and IHC assays, our results indicated that the rate of *ALK* false positive or false negative from RT‐ddPCR, which was determined using an automatic operation and data analysis system, probably would be less than those from FISH or IHC and deserving to be explored in a large sample size.^23^

In conclusion, we developed and validated a novel fusion‐induced asymmetric transcription assay‐based reverse transcription droplet digital PCR system with reliable highly clinical sensitivity, specificity, and accuracy for *ALK* rearrangement detection on FFPE samples from NSCLC. This new method would be a potential alternative for conventional *ALK* assessment approaches in routine clinical work, particularly liquid biopsy, after verification in the near future because of its high sensitivity, exact quantitative measures, low‐cost, ease of use and automatic result evaluation.

## Disclosure

All authors declare that they have no conflict of interest to disclose.
